# Endovascular Treatment of a Dissected Celiac Trunk Aneurysm Complicated with Consequent Pseudoaneurysm: Primary Treatment and Treatment Relapse after 5 Years

**DOI:** 10.1155/2015/291953

**Published:** 2015-06-01

**Authors:** Francesco Giurazza, Mattia Silvestre, Amedeo Cervo, Franco Maglione

**Affiliations:** ^1^Department of Radiology, Università Campus Bio-Medico di Roma, Via Alvaro Del Portillo 200, 00100 Rome, Italy; ^2^Department of Radiology, Università degli Studi Federico II di Napoli, Via Pansini 5, 80100 Naples, Italy; ^3^Department of Vascular Radiology, A.O.R.N. Antonio Cardarelli, Via A. Cardarelli 9, 80100 Naples, Italy

## Abstract

We report on an asymptomatic 56-year-old male with incidental diagnosis of celiac trunk aneurysm, diagnosed during an ultrasound scan performed to control polycystic kidney disease. The CT scan revealed a 3.8 cm saccular aneurysm of the celiac artery dissected in the superior wall with a consequent 4.3 cm pseudoaneurysm; we adopted an endovascular approach to exclude the lesion by catheterizing the celiac trunk and positioning a vascular plug in the common hepatic artery and a covered stent in the splenic artery; finally we fulfilled the aneurysm sac with Onyx. 30-day control CT scan revealed procedural success. Five years later he came back to our department for an aneurysm relapse in the common hepatic artery. We performed a second endovascular approach with a superselective catheterization of the pancreaticoduodenal arcade in order to exclude the lesion with Onyx and microcoils. Nowadays the patient is in good clinical conditions. Endovascular approach is a valuable method to treat visceral aneurysms; however, long-term imaging follow-up is essential to monitor the risk of relapse.

## 1. Introduction

Aneurysms of celiac trunk are uncommon and represent only 4% of all the visceral aneurysms [1].

To avoid their rupture, treatment is indicated in patients with aneurysms >20 mm [2].

Spontaneous dissection of these lesions is even more rare and, in presence of a consequent pseudoaneurysm, there is the indication for immediate treating because of the significant risk of hemorrhage.

Open surgery is the classical treatment, but it has a postoperative mortality rate of 5% [3].

The endovascular approach reduces the postoperative complications and the duration of the hospital stay and is replacing open surgery as the preferred surgical option, at least in selected cases [4].

We report here on a 56-year-old patient affected by a dissected saccular aneurysm of the celiac trunk with a consequent pseudoaneurysm treated by endovascular technique.

## 2. Case Presentation

In June 2009 a 56-year-old male affected by polycystic kidney disease (PKD) came to our attention because during a routinary renal ultrasound (US) control it was diagnosed incidentally as an aneurysm of the celiac trunk.

**Figure 1:**
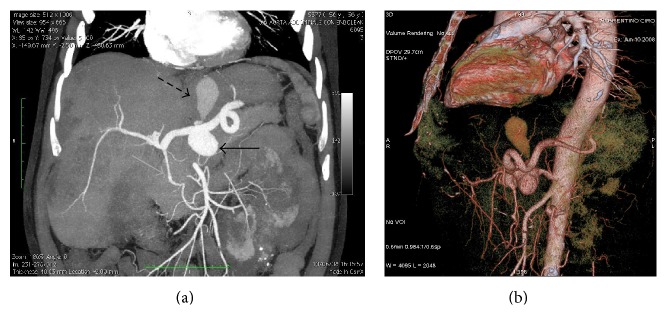
CT scan. (a) Maximum intensity projection (MIP) reconstruction of the arterial phase in the coronal plane showing a 3.8 cm aneurysm of the celiac trunk (solid black arrow) with a 4.3 cm pseudoaneurysm (dashed black arrow) originating from the dissection of the superior margin of the aneurysm sac. An ectasic pancreaticoduodenal arcade is highlighted (grey arrow). (b) Volume rendering (VR) reconstruction in the sagittal plane showing aneurysm and pseudoaneurysm.

The computed tomography (CT) examination (Figures 1(a) and 1(b)) detected a 3.8 cm saccular aneurysm of the celiac trunk dissected in the cranial portion of the lesion with a consequent 4.3 cm pseudoaneurysm; in the same exam, an ectatic pancreaticoduodenal arcade and a small aneurysm of the left renal artery (1.3 cm) were detected too.

The patient was asymptomatic and did not refer any abdominal trauma. Because of PKD, he suffered from chronic renal failure, with no other relevant pathologies in anamnesis.

Because of the significant risk of rupture [5], we decided on an endovascular approach.

We practiced a bilateral femoral access.

**Figure 2:**
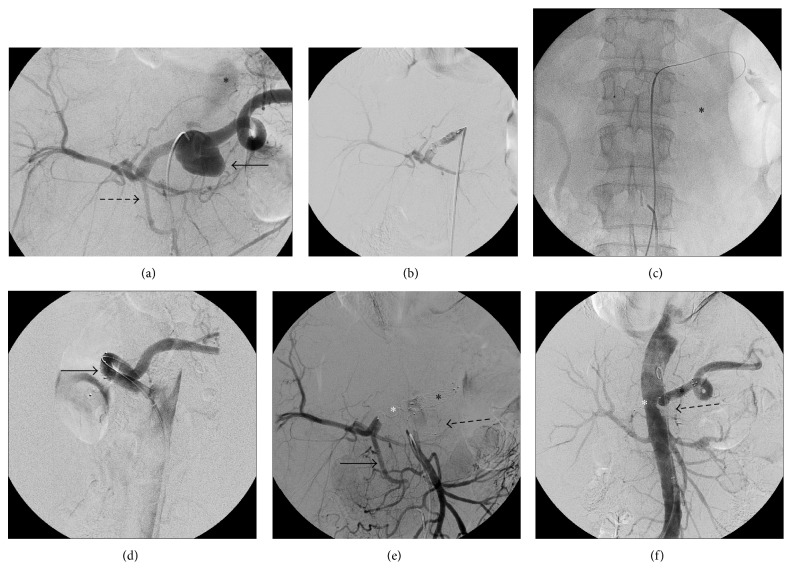
Diagnostic and procedural angiography. (a) Contrast injection from the celiac trunk confirming CT scan report: aneurysm of the celiac trunk (black arrow), cranial dissection, and consequent pseudoaneurysm (asterix) presenting with delayed enhancement and ectatic pancreaticoduodenal arcade (dashed black arrow); (b) release of a 12 mm vascular plug in the proximal third of the common hepatic artery; ((c)-(d)) positioning of a microcatheter (asterix) inside the aneurysm sac and of a guidewire in the splenic artery for the following release of a 12 × 80 mm covered stentgraft (black arrow); (e) contrast injection from the superior mesenteric artery showing the pancreaticoduodenal arcade (solid black arrow) supporting liver vascularization after occlusion of the hepatic artery with a vascular plug (white asterix), stent in the splenic artery (black asterix), and microcatheter inside the aneurysm sac (dashed black arrow); (f) aortography showing the complete exclusion of the aneurysm sac fulfilled with Onyx (dashed black arrow), patency of the covered stent positioned in the splenic artery (black asterix), and occlusion of the hepatic artery with a vascular plug (white asterix).

Initially we performed a diagnostic angiography that confirmed the CT findings (Figure 2(a)).

First we catheterized the celiac trunk and we released a 12 mm Amplatzer Vascular Plug II (St. Jude Medical) in the proximal third of the common hepatic artery (Figure 2(b)).

Then, we positioned a 2.4-French (Fr) microcatheter inside the aneurysm sac in order to fulfill the aneurysmatic lesion with a liquid embolic agent, that is, Onyx 34 (ev3-Covidien) (Figure 2(c)); before delivering Onyx, we placed a 12 × 80 mm covered stentgraft (Fluency, Bard Peripheral Vascular) in the splenic artery with the proximal extremity in the aorta in order to preserve the spleen vascularization (Figure 2(d)).

After stent positioning, we filled the aneurysm sac with Onyx 34.

The final controls showed exclusion of the aneurysm sac with patency of the splenic artery and hepatic vascular flow sufficiently supported by the pancreaticoduodenal arcade (Figures 2(e) and 2(f)).

**Figure 3:**
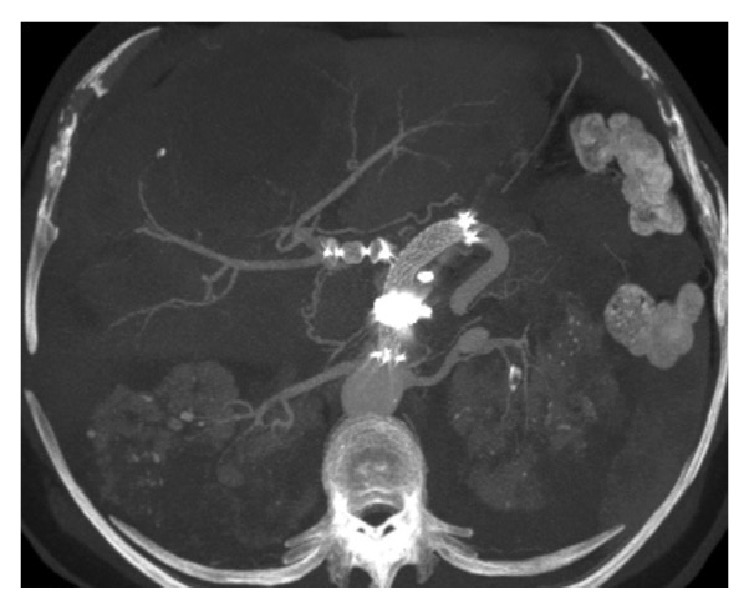
CT scan performed 30 days after the procedure confirming procedural success. MIP reconstruction: complete exclusion of the aneurysm with patency of the Fluency stent in the splenic artery and occlusion of the common hepatic artery with Amplatzer Vascular Plug and Onyx to fill the aneurysm sac. A 1.3 cm of the left renal artery is recognizable.

The CT scan performed 30 days after the intervention confirmed the procedural success (Figure 3).

**Figure 4:**
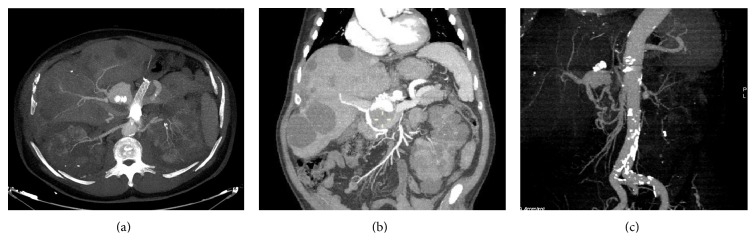
CT scan after 5 years from intervention. (a) MIP reconstruction in the axial plane showing aneurysm relapse in the hepatic artery; patency of the stent in the splenic artery is also evident; MIP (b) and VR (c) reconstructions in coronal and sagittal oblique planes demonstrating the pancreaticoduodenal arcade supplying the aneurysm relapse; the vascular plug, previously positioned, is noted. Renal and hepatic cysts are observable in Figure (b).

Five years later the patient came back to our department because of disease relapse; he stood in good clinical conditions without symptoms; however, his last CT scan revealed again a 3 cm aneurysmatic lesion in correspondence with the previous site of treatment of the common hepatic artery (Figure 4). The left renal aneurysm was unchanged.

This new lesion was supplied retrogradely by the pancreaticoduodenal arcade.

**Figure 5:**
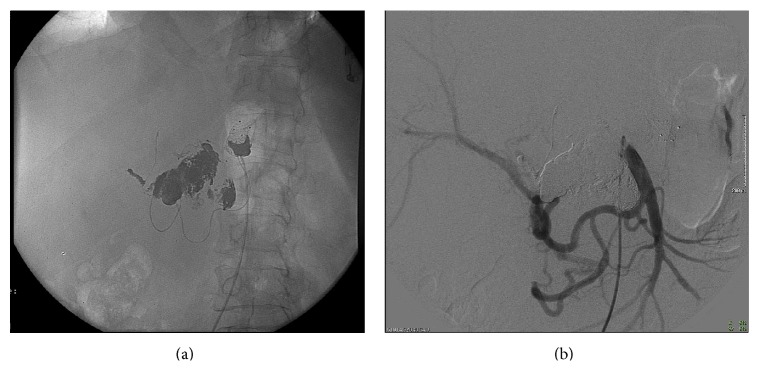
Diagnostic and procedural angiography. Approach from superior mesenteric artery catheterization. (a) Superselective microcatheterization of the pancreaticoduodenal arcade to release Onyx and microcoils into the relapsed aneurysm sac; small fragments of Onyx are appreciable in the left hepatic artery because of involuntary migration; (b) final injection control showing complete exclusion of the lesion with preserved hepatic vascular flow.

So, we performed a new endovascular approach: we catheterized the superior mesenteric artery and the pancreaticoduodenal arcade with a 2.7 Fr microcatheter in order to reach the hepatic artery retrogradely; finally we embolized the sac with Onyx 34 and 8 mm microcoils (MicroNester, Cook Medical) (Figures 5(a) and 5(b)).

The final control showed the good procedural outcome with complete exclusion of the aneurysmatic sac and preserved patency of the main hepatic vascular flow; small fragments of Onyx migrated in the left hepatic artery without consequences.

Nowadays the patient is in good clinical conditions.

## 3. Discussion

Visceral artery aneurysms are rare and the most common district involved is the splenic artery (60%). In approximately 40% of the cases the pathology is polidistrectual and in 20% of the cases is associated with abdominal aorta aneurysm [6].

Celiac trunk involvement is reported only in 4% of the subjects [1] and in many cases patients affected are asymptomatic [7].

Regardless of the presence of symptoms, diagnosis is based on US and/or CT scan.

Risk of rupture is significant when the diameter of the aneurysm is greater than 20 mm and so this is the main indication for treatment [8]; indeed the mortality of ruptured aneurysm is between 40% and 100% [9].

This kind of lesion can be approached by conventional surgery or endovascular treatment.

The choice depends mainly on general and local conditions, that is, patient comorbidities, aneurysm size, interventional radiology expertise, and so forth.

Our patient was asymptomatic and with polidistrectual disease (aneurysms of the celiac trunk and of the left renal artery); because of the elevated risk of rupture due to size and dissection with the consequent formation of a pseudoaneurysm, we decided to treat this patient. The unique relevant pathologies in anamnesis were PKD and chronic renal failure; our patient performed also a skin biopsy in order to assess the presence of collagen disease but results were negative.

Different endovascular treatments of dissected celiac trunk aneurysms have been reported [10–12] but from literature data a standard approach does not emerge.

In any case the goal of the treatment should be to exclude the aneurysm sac preserving the vascularization to the abdominal organs. This can be reached thanks to the arterial network of the abdominal viscera which is reach of vascular anastomosis, permitting embolizing several vessels without ischaemic damage.

In order to take advantage of this, an accurate preprocedural planning is essential; this should be performed on CT scan using the multiple postprocessing reconstruction software commonly available in radiology departments.

We focused on the ectatic pancreaticoduodenal arcade and this allowed us to embolize the hepatic artery without risk of ischaemic liver damage; in order to preserve the splenic vascularization, we decided to release a covered stent in the splenic artery. This permitted also injection of embolizing materials without risk of migration.

We chose to embolize the aneurysm sac with liquid embolic agent because of the size of the lesion; we injected Onyx 34 because it presents a safer control in comparison to glue avoiding distal migration, due to the close position of the microcatheter into the lesion.

Embolizing the aneurysm, the pseudoaneurysm was excluded as well, as demonstrated by the 30-day control CT.

The patient came back to our department 5 years later, after performing a CT scan presenting disease relapse with a new aneurysm sac refurnished with arterial flow coming from the pancreaticoduodenal arcade. So, we embolized the new lesion with both coils and Onyx. The migration of small fragments of Onyx did not imply any risk for the liver vascularization; however, if possible, it should be avoided by gently injecting the embolic agent.

We choose not to treat the left renal artery aneurysm because of its small size (1.3 cm); indeed the last CT scan showed no lesion changes.

On the basis of our knowledge, this is the first case reported in the literature of a celiac trunk aneurysm dissected with a consequent pseudoaneurysm.

## 4. Conclusion

Endovascular approach is a valuable technique to treat visceral aneurysms.

The lesion size and the presence of complications, that is, dissection, pseudoaneurysm, and so forth, represent a strong indication to treatment.

However, a strict and continuous imaging follow-up must be performed to monitor possible disease relapse.
